# MicroRNA Let-7i Is a Promising Serum Biomarker for Post-stroke Cognitive Impairment and Alleviated OGD-Induced Cell Damage *in vitro* by Regulating Bcl-2

**DOI:** 10.3389/fnins.2020.00215

**Published:** 2020-03-24

**Authors:** Zhan-Qiang Wang, Kuo Li, Jie Huang, Tian-Tian Huo, Pei-Yuan Lv

**Affiliations:** ^1^Department of Neurology, Hebei Medical University, Shijiazhuang, China; ^2^Department of Neurology, Hebei General Hospital, Shijiazhuang, China; ^3^Department of Neurology, Cangzhou People’s Hospital, Cangzhou, China; ^4^No. 2 Department of Neurology, Cangzhou Central Hospital, Cangzhou, China

**Keywords:** miR-let-7i, microRNA, post-stroke cognitive impairment, Bcl-2, cell damage, oxygen–glucose deprivation

## Abstract

**Background:**

The mechanism of post-stroke cognitive impairment (PSCI) has not been explained. We aimed to investigate whether miR-let-7i participates in the PSCI and illuminates its underlying role in oxygen–glucose deprivation (OGD)-induced cell apoptosis.

**Methods:**

Blood samples from 36 subjects with PSCI and 38 with post-stroke cognitive normality (Non-PSCI) were collected to evaluate the differential expression of miR-let-7 family members, using qRT-PCT analysis. Spearman correlation was performed to estimate the correlation between the miR-1et-7i level and Montreal Cognitive Assessment (MoCA) score. Treatment of SH-SY5Y cells with OGD was used to induce cell apoptosis *in vitro*. Effects of miR-let-7i on OGD-induced cell apoptosis was estimated after transfection. The target gene of miR-let-7i was analyzed by dual luciferase reporter gene assay.

**Results:**

The expression of miR-let-7i was up-regulated in PSCI patients compared with Non-PSCI (*p* < 0.001) and negatively correlated with MoCA score (*r* = −0.643, *p* < 0.001). When exposed to OGD, SH-SY5Y cells showed significant apoptosis accompanied by miR-let-7i up-regulation. In OGD-treated cells, miR-let-7i up-regulation was accompanied by cell apoptosis, while down-regulation showed the opposite effect. Luciferase reporter assay showed that Bcl-2 was a target gene of miR-let-7i. Western blot showed that miR-let-7i up-regulation promoted Bcl-2 expression, while qRT-PCR showed that miR-let-7i had no effect on Bcl-2 expression.

**Conclusion:**

miR-let-7i was overexpressed in PSCI patients and it could be used as a diagnostic biomarker for PSCI. We illuminated the potential mechanism that miR-let-7i alleviated OGD-induced cell damage by targeting Bcl-2 at the post-transcriptional level.

## Introduction

Stroke is a major cause of cognitive impairment and dementia and has been reported to increase the risk of cognitive impairment at least five to eight times (Merino, 2002; Srikanth et al., 2003; Qu et al., 2015). At present, the prevalence of post-stroke cognitive impairment (PSCI) is increasing because of the aging population and a rise in the number of stroke survivors (Jacquin et al., 2014). The prevalence of PSCI in various countries was varied from 17% to 92%, and it has also reached 80.97% in China (Pasi et al., 2012; Qu et al., 2015). PSCI occur immediately after a stroke or after a certain period, but it is often overlooked at onset (Chi et al., 2019). Consequently, the timely diagnosis and prevention of PSCI are critical at present. However, there is a lack of biomarkers that could accurately predict PSCI. Previously, the pathogenesis of PSCI has been shown that due to the paucity of energy or oxygen to the brain, the region-specific neural damage occurred, ultimately leading to a progressive cognitive impairment (Wahul et al., 2018). Even so, the pathogenesis of PSCI remains unclear.

MicroRNAs (miRNAs) are a class of small endogenous RNA molecules that regulate gene expression in many biological processes (Brown and Naldini, 2009; Keasey et al., 2016). MiRNAs presented in human serum in a highly stable form that could be resistant to repeated freeze–thaw cycles and endogenous enzymatic degradation (Scholer et al., 2010). Meanwhile, miRNA expression levels are consistent across individuals of the same species (Reid et al., 2011). Because of these properties, miRNAs have become a popular diagnostic marker. Previously, miR-132 was demonstrated to be a risk marker of PSCI and could be used as a diagnostic biomarker for PSCI (Huang et al., 2016). Recently, Balakathiresan et al. (2012) analyzed the expression of various candidate miRNAs in the serum of animals post-blast overpressure injury. Among these, miR-let-7i was reported to be highly enriched in the brain of rats with traumatic brain injury (TBI). In experimental brain injury, miR-let-7i is up-regulated in cerebrospinal fluid as early as 3 h post-injury and has been used as a diagnostic biomarker for TBI (Bhomia et al., 2016). Thus, we speculated that miR-let-7i may be used as an alternative biomarker for PSCI. However, the role of miR-let-7i in the pathogenesis of PSCI has not yet been elaborated, especially its molecular mechanism. It is well acknowledged that hypoxia could induce oxidative stress, which is involved in neuronal cell death, which is one of the leading causes of neurodegenerative diseases, such as cerebral ischemia reperfusion after stroke (Bains and Shaw, 1997; Tabner et al., 2005). Thus, investigating the role of miR-let-7i in the prevention of neuronal cell death has the potential to prevent PSCI.

The aim of present study was to investigate whether miR-let-7i participates in the pathogenesis of PSCI and illuminates its underlying role in oxygen–glucose deprivation (OGD)-induced cell apoptosis *in vitro*. In present study, we collected blood samples from 36 subjects with PSCI and 38 with Non-PSCI to detect the differential expression of miR-let-7 family members and evaluate the relationship between miR-let-7 and PSCI. Importantly, we induced apoptosis by OGD treatment *in vitro* to simulate brain injury, expecting to elucidate the molecular mechanism of miR-let-7 in PSCI.

## Materials and Methods

### Human Subjects

In this study, blood samples were collected from 38 subjects with post-stroke cognitive normality (Non-PSCI) and 36 subjects with PSCI. Blood samples from the peripheral vein of subjects were collected in tubes with EDTA anticoagulant. After centrifugation of the blood samples, plasma (280 μl) was collected for quantitative real-time polymerase chain reaction (qRT-PCR) analysis using small nuclear RNA U6 as internal control. The study was approved by the Ethics Committee of the Cangzhou Center Hospital and Cangzhou People’s Hospital. Written informed consent was obtained from each participant.

The inclusion criteria of PSCI group were as follows: (1) the subjects had a history of stroke and/or neuroimaging (CT or MRI) provided evidence of cerebrovascular disease; (2) cognitive impairment was judged to have a vascular cause, and a Montreal Cognitive Assessment (MoCA) score <26 was obtained (1-point correction for persons educated no more than 12 years); (3) the stroke in question was the patient’s first and occurred no more than 1 year prior to the study; and (4) the patient was conscious with stable vital signs (Nasreddine et al., 2005; Carson et al., 2018). The inclusion criteria of the Non-PSCI group were the same as those of the PSCI patients, with the exception that the MoCA score was greater than 26.

The exclusion criteria of PSCI group were as follows: (1) patients with other diseases that cause cognitive impairment, such as brain tumor, brain trauma, or brain parasitic disease; (2) patient had long exhibited cognitive impairment or had used drugs related to cognitive impairment prior to stroke; (3) patients with signs of serious speech, vision, hearing impairment, or mental disorders that influence cognitive examination; (4) Beck Depression Inventory Score >13; and (5) alcohol or drug abuse, heart failure, respiratory failure, or other organ failure; and pregnant and lactating women.

### Cell Culture and OGD Treatment

Human neuroblastoma cell line SHSY-5Y was originally obtained from Chinese Academy of Medical Sciences (Beijing, China). Cells (density: 1 × 10^4^ cells/ml) were maintained in DMEM medium supplemented with 10% fetal bovine serum (FBS, Invitrogen, United States), 100 U/ml penicillin, and 100 μg/ml streptomycin in an incubator at 37°C with a 5% CO_2_ atmosphere.

Oxygen–glucose deprivation was performed as described previously (Lee et al., 2011). Briefly, on the second day of differentiation, cell culture DMEM medium was removed and was replaced by the glucose-free DMEM medium before OGD treatment. Then, cells were cultured in an anaerobic, temperature-controlled (37 ± 0.5°C) chamber that was flushed with 5% CO_2_ and 95% N_2_ (v/v) for 30 min to remove residual oxygen. Cells were incubated in this solution at 37°C for a 4-h period to produce lethal oxygen deprivation. For reoxygenation, the culture medium was refreshed with normal DMEM medium, and then cells were returned into the humidified incubator and reoxygenated for 24 h. The normal medium under normoxia served as control. Cells were collected and analyzed immediately at 24 h after reoxygenation.

### Cell Viability Assay and Apoptosis Assay

Cell Counting Kit-8 was used to measure the cell viability according to the manufacturer’s protocol (Beyotime, China). SHSY-5Y cells were seeded in the 96-well plates at a density of 5 × 10^3^ cells/well. After the incubation, the culture medium was replaced by basal medium containing 10% CCK-8 solution, and the incubation continued for another 1 h at 37°C. Absorbance was measured at 450 nm by a microplate reader (Bio-Rad, United States).

Flow cytometry assay was performed to measure the cell apoptosis. Cells were suspended in 100 μl of binding buffer (Nanjing KeyGen Biotech. Co., Ltd., China). Then, apoptosis was detected using Hoechst 33342/PI Kit according to the manufacturer’s protocol (Solarbio, Beijing, China). Cells were stained with 5 μl of Hoechst 33342 solution and incubated in the dark at room temperature for 10 min. Then, 5 μl of propidium iodide (PI) was added to the cell suspension. Hoechst 33342/PI-stained cells were analyzed immediately by using Scalibur Flow Cytometer (BD Biosciences, United States).

### siRNA Transfection

The miR-let-7i mimics, miR-let-7i inhibitors (anti-miR-let-7i), and their corresponding negative controls were chemically synthesized by Shanghai GenePharma Co., Ltd. (Shanghai, China). siRNA transfection was performed with Superfect^TM^ Transfection Reagent (Qiagen, United States) according to the manufacturer’s instruction. Cells were transfected with 50 nM oligonucleotides and then incubated for 48 h. The overexpression and inhibition of miR-let-7i were confirmed by RT-PCR after transfection.

### Quantitative Real-Time Polymerase Chain Reaction

Total RNA was isolated using TRIzol reagent (Invitrogen, United States) according to the manufacturer’s instruction. For miR-let-7i detection, cDNA was synthesized using One Step PrimeScript miRNA cDNA Synthesis Kit (TaKaRa, China). For Bcl-2 detection, cDNA was synthesized using PrimeScript RT Reagent Kit (TaKaRa, China). The mRNA expression levels were detected with SYBR Green RT-PCR Kit (Takara Bio, Japan). Then, the qRT-PCR analysis was performed in triplicate on an ABI 7500 Fast Real-Time PCR System (Applied Biosystems, United States), using small nuclear RNA U6 and GAPDH as the endogenous control for miR-let-7i and Bcl-2, respectively. Data were calculated by the comparative cycle threshold (CT) (2^–ΔΔ*CT*^) method. The PCR primer sequences were as follows.

(1) miR-let-7i forward: 5′-TGAGGTAGTAGTTTGTGCTG TT-3′; U6, forward primer: 5′-TGCGGGTGCTCGCT TCGGCAGC-3′. The reverse primers for miR-let-7i and U6 were universal adaptor primers available in a ready-to-go format in the One Step PrimeScript miRNA cDNA Synthesis Kit (D350A; Takara, China).
(2) Bcl-2 forward: 5′-GGTGGGGTCATGTGTGTGG-3′; reverse: 5′-CGGTTCAGGTACTCAGTCATCC-3′;
(3) GAPDH forward: 5′-CTGGGCTACACTGAGCACC-3′; reverse: 5′-AAGTGGTCGTTGAGGGCAATG-3′.

### Western Blot

Expression of Bcl-2 was detected by Western blot using specific primary antibody to Bcl-2 (Santa Cruz, United States). The GADPH was used as an endogenous control. Whole-cell lysates were extracted using radioimmunoprecipitation assay lysis buffer for 30 min on ice (Thermo Fisher Scientific, United States). Protein concentration was quantified with Bio-Rad protein assay reagent (Bio-Rad, United States). Then, equal amounts of proteins (40 μg) of each sample were separated on 10% sodium dodecyl sulfate–polyacrylimide gel electrophoresis (SDS–PAGE), and then were transferred onto the polyvinylidene fluoride (PVDF) membrane (Millipore, United States). Subsequently, PVDF membrane was blocked in skim milk at 25°C for 2 h and subsequently incubated at 4°C overnight with primary monoclonal antibodies against Bcl-2 or GAPDH (all in 1:1000 dilution, Santa Cruz, CA, United States). PVDF membrane was washed three times in Tris-buffered saline with Tween-20 (TBST) containing 5% defatted milk, and then was incubated with horseradish peroxidase-conjugated (HRP)-linked secondary IgG antibodies (1:1000 dilution, Santa Cruz, CA, United States) for 1 h at room temperature. The bands of bound secondary antibody were detected with an enhanced chemiluminescence kit (Pierce Biotechnology, United States), and the signals were detected with a SuperSignal Protein Detection kit (Pierce Biotechnology, United States). The band intensity of specific proteins was quantified subsequent to normalization with the density of GAPDH using ImageJ (National Institutes of Health, United States).

### Target Prediction and Dual Luciferase Reporter Gene Assay

Based on bioinformatic prediction (Targetscan^1^), BCL-2 was selected as the candidate target of miR-let-7i. Dual luciferase reporter gene assay was preformed to verify the targeting. Briefly, gaussia luciferase (GLuc) assay and Phospha-Light kits (Applied Biosystems, United States) were used for luciferase and secreted alkaline phosphatase (SEAP) assay according to the manufacturers’ instructions. SHSY-5Y cells (1 × 10^5^ cells/well) were cultured in 24-well plates in combination with a standard dual reporter expression plasmid system, which was consisted of a GLuc reporter gene and SEAP with a copy of the 3′-UTR sequence of BCL-2. A mutant version of the vector was used as a negative control, which was prepared by modifying the seed sequence and confirmed by sequencing. After transfection for 24 h, the luciferase activity was determined by dual-luciferase reporter assay system (Promega, United States) according to the manufacturer’s protocol.

### Statistical Analysis

Statistical analysis was performed by SPSS software version 18.0 (SPSS, Inc., United States). Quantitative data from clinical samples were described using mean ± standard deviation (SD), and qualitative data were described by number and frequency. The normality of the data distribution was tested with Kolmogorov–Smirnov test, and the distribution of the data was considered normal. The results from cell experiments were presented as mean ± standard error (SE) of three separate experiments. Student’s *t*-test or chi-square test was used to perform the between-group comparisons when appropriate. For multiple group comparison, the difference was detected by one-way ANOVA. Spearman correlation was performed to estimate the correlation between the miR-1et-7i level and MoCA score. Diagnostic performance of the miR-1et-7i was assessed by constructing a receiver operating characteristic (ROC) curve and evaluated by calculating the area under curve (AUC). For *in vitro* experiments, results were expressed as mean ± SE of at least three separate experiments. All statistical tests were two-sided, with significance set at *p* < 0.05 along with 95% confidence intervals (CIs).

## Results

### Patient Characteristics and the Differential Expression of miR-let-7 Family Members

**FIGURE 1:**
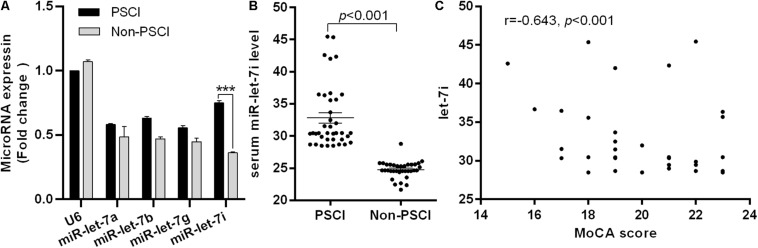
Differential expression of miR-let-7i in PSCI patients. **(A)** The differential expression of miR-let-7 family member between PSCI and Non-PSCI patients. **(B)** The serum level of miR-let-7i in PSCI and Non-PSCI patients. **(C)** Spearman correlation analysis showed a negative correlation between the serum miR-let-7i level and MoCA scores. PSCI, post-stroke cognitive impairment; Non-PSCI, post-stroke cognitive normality; MoCA, Montreal Cognitive Assessment. ***p < 0.001.

To explore the potential biomarkers in the miR-let-7 family for patients with PSCI, blood samples were collected from 38 patients with Non-PSCI and 36 patients with PSCI to detect the miRNA expression level. The demographic information and biochemical data of included patients are shown in Table 1. The two groups were generally well balanced for age, gender, disease history, and biochemical data (*p* > 0.05). As shown in Figure 1A, miR-let-7i was significantly up-regulated in patients with PSCI compared to patients with Non-PSCI (*p* < 0.001). However, the other miRNAs, including miR-let-7a, miR-let-7b, and miR-let-7g, did not exhibit the differential expression between Non-PSCI and PSCI patients. Thus, miR-let-7i was selected as a potential biomarker to verify its relationship with the PSCI.

**TABLE 1:** Baseline characteristics of patients.

Baseline characteristics	PSCI patients (*n* = 36)	Non-PSCI patients (*n* = 38)	*P*-value
Gender, (M/F)	16/20	15/23	0.27
Age (years)	68 (57–72)	67 (52–66)	0.12
Time from symptoms onset to hospital admission (days)	3 (1–5)	3 (1–7)	0.34
Years of education (years)	12 (3–16)	9 (4–16)	0.74
BMI (kg/m^2^)	24.6 (22.1–26.0)	24.8 (21.7–26.3)	0.69
Diabetes mellitus, *n* (%)	12 (33.3)	11 (28.9)	0.91
Hyper lipidemia, *n* (%)	12 (33.3)	13 (34.21)	0.57
Heart disease, *n* (%)	10 (27.7)	11 (28.9)	0.67
Alcohol consumers, *n* (%)	12 (33.3)	14 (36.8)	0.78
Smoking, *n* (%)	12 (33.3)	13 (34.21)	0.70
WBC (× 10^9^/L)	4.5 ± 1.3	5.1 ± 1.7	0.91
HbA1C (%)	5.8 ± 0.3	5.9 ± 0.4	0.78
Cys-C (mg/ml)	1.2 ± 0.03	1.1 ± 0.02	0.67
Small vessel occlusion, *n* (%)	23 (63.9)	25 (65.7)	0.36
NHISS score at admission	5 (2–6)	5 (2–5)	0.61
MoCA score	19 ± 2.6	27 ± 1.5	0.005

*BMI, body mass index; WBC, white blood cell; HbA1C, hemoglobin A1c; Cys-C, cystatin-c; NHISS, National Institute of Health Stroke Scale; MoCA, Montreal Cognitive Assessment.*

### Relationship Between miR-let-7i and PSCI

We further measured the serum levels of miR-let-7i in Non-PSCI and PSCI patients and verified that the serum levels of miR-let-7i in PSCI patients were significantly higher than that of Non-PSCI patients (*p* < 0.001, Figure 1B). Then, we analyzed the correlation between the cognitive function (MoCA score) and the serum level of miR-let-7i using Spearman correlation analysis. The results showed that the relative expression of miR-let-7i was negatively correlated with MoCA score (*r* = −0.643, *p* < 0.001, Figure 1C). Overall, the above results suggested that miR-let-7i may be involved in the pathogenesis of PSCI.

### Diagnostic Performance of miR-let-7i for PSCI

**FIGURE 2:**
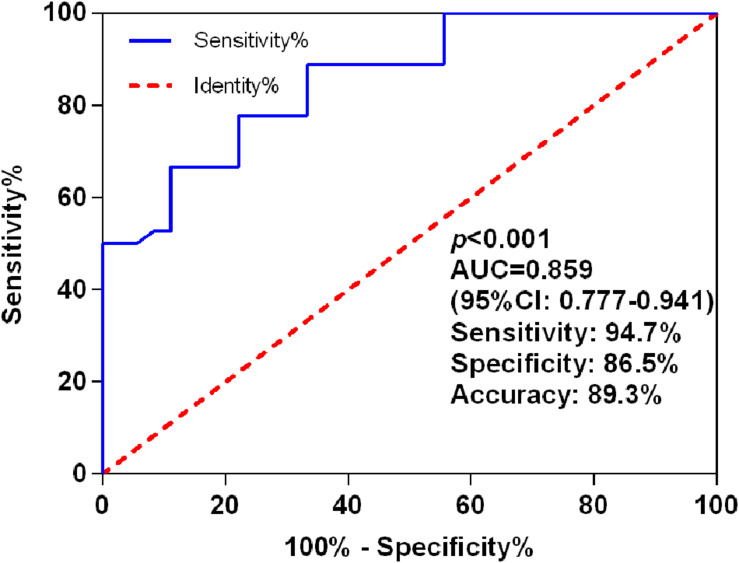
ROC curves for miR-let-7i levels to discriminate PSCI patients from post-stroke cognitive normality participants. ROC, receiver operating characteristic; AUC, area under curve; PSCI, post-stroke cognitive impairment.

The AUC for miR-let-7i level in discrimination between PSCI patients and Non-PSCI was 0.859 (95% CI, 0.777–0.941; *p* < 0.001), with a sensitivity of 94.7%, a specificity of 86.5%, and an accuracy of 89.3% (Figure 2). These results indicated that miR-let-7i would be used as a diagnostic biomarker for PSCI.

### miR-let-7i Alleviated OGD-Induced Cell Apoptosis *in vitro*

**FIGURE 3:**
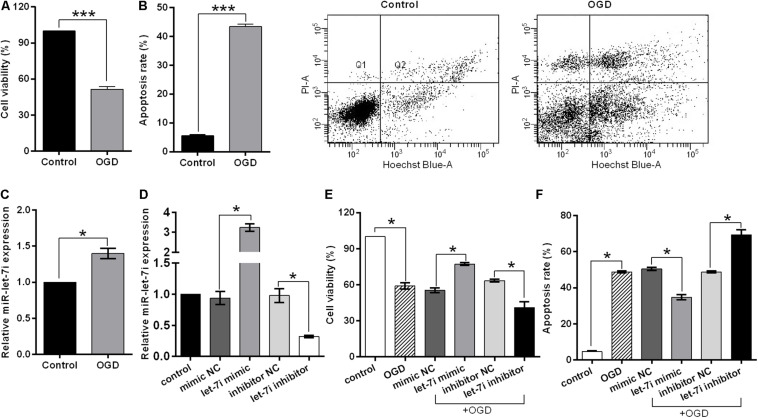
miR-let-7i alleviated OGD-induced cell apoptosis *in vitro*. SHSY-5Y cells were exposed to OGD, and then ell viability **(A)**, apoptosis rate **(B)**, and miR-let-7i expression **(C)** were measured by CCK-8 assay, flow cytometry assay, and qRT-PCT, respectively. **(D)** SH-SY5Y cells were transfected with minic-NC, miR-let-7i mimic, inhibitor NC, or miR-let-7i inhibitor, and non-transfected cells were used as control. Expression of miR-let-7i was measured by qRT-PCR. SH-SY5Y cells were transfected and then cell viability **(E)** and apoptosis rate **(F)** were measured by CCK-8 assay after OGD treatment. OGD, oxygen glucose deprivation. Data presented are the mean ± standard error (*n* = 3). *p < 0.05, ***p < 0.001.

In order to simulate the cell damage *in vitro*, SH-SY5Y cells were exposed to OGD treatment. Cell viability and apoptosis rate were used to assess the cell injury. We observed that OGD significantly inhibited the cell viability (*p* < 0.001, Figure 3A), while it enhanced the apoptosis rate compared to the control (*p* < 0.001, Figure 3B). Subsequently, to evaluate whether miR-let-7i is involved in OGD-induced cell apoptosis, we detected the expression of miR-let-7i after OGD treatment. As shown in Figure 3C, the expression of miR-let-7i was significantly up-regulated compared to the control (*p* < 0.05), indicating that the expression of miR-let-7i is associated with OGD-induced cell apoptosis *in vitro*.

To further verify the role of miR-let-7i in the OGD-induced cell apoptosis *in vitro*, we next examined its effect on cell viability in stable transfection cell lines of miR-let-7i or its inhibitor. After transfection, miR-let-7i levels were significantly up-regulated in stable transfected cells of miR-let-7i, while they were down-regulated in transfection cells of miR-let-7i inhibitor compared to the controls (*p* < 0.05, Figure 3D). These results showed that the alterations in expression level of miR-let-7i were achieved successfully. Next, the cell viability and apoptosis were evaluated after transfection in transfected cell lines. When exposed to OGD treatment, the cell viability could be partly amplified by up-regulating miR-let-7i expression, but were attenuated by miR-let-7i up-regulation (*p* < 0.05, Figure 3E). On the contrary, the OGD-induced cell apoptosis could be partly attenuated by miR-let-7i up-regulation, but were amplified with miR-let-7i down-regulation (*p* < 0.05, Figure 3F). Overall, these results suggested that miR-let-7i exerted an anti-apoptosis effect, thus alleviating the OGD-induced cell apoptosis *in vitro*.

### Bcl-2 Is a Post-transcriptional Target Gene of miRNA-let-7i

**FIGURE 4:**
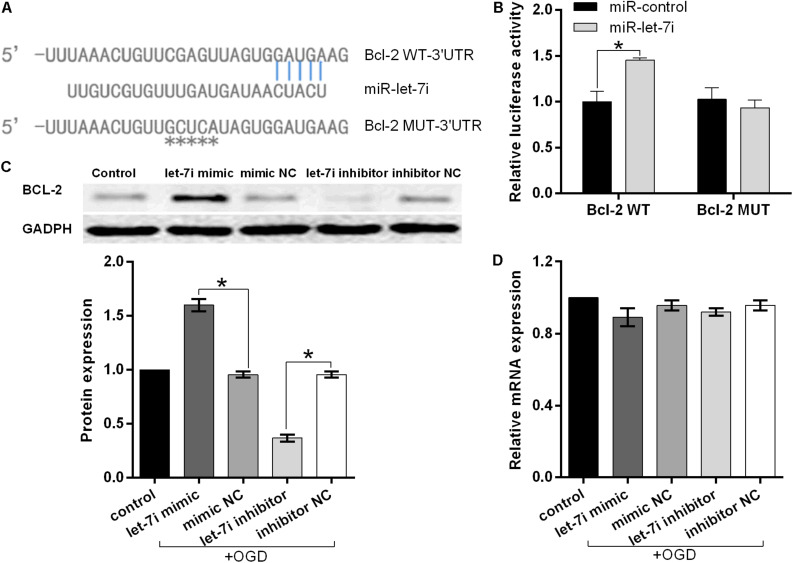
Bcl-2 is the direct target of miRNA-let-7i and positively regulated by miRNA-let-7i at the post-transcriptional level. **(A)** Alignment of miR-let-7i with Bcl-2 3′-UTR sequences. **(B)** Relative luciferase activity of reporters containing wild-type or mutated type with miR-let-7i target sites. Transfected and non-transfected cells were exposed to OGD, and the expression of Bcl-2 protein **(C)** and mRNA **(D)** was measured by Western blot and qRT-PCT, respectively. Data presented are the mean ± standard error (*n* = 3). *p < 0.05.

To further investigate the molecular mechanism underlying miR-let-7i-mediated anti-apoptosis *in vitro*, a vast bioinformatic analysis through bioinformatics software Targetscan (see text footnote 1) was performed to examine putative targets of the miRNA. Among the candidates, the seed sequence of miR-let-7i is complementary to that of Bcl-2 3′-UTR (Figure 4A). Moreover, this putative binding was verified by dual luciferase reporter gene assay. The miR-let-7i co-transfection significantly enhanced the activity of luciferase reporter containing the wild-type 3′-UTR of Bcl-2 (*p* < 0.05), while it had no effect on the activity of the vector containing mutational 3′-UTR of Bcl-2 (Figure 4B). Overall, these results indicated that Bcl-2 is the target of miR-let-7i.

To further confirm the regulatory relationship between miR-let-7i and Bcl-2, we examined the expression of Bcl-2 at both mRNA and protein levels in transfected cells after OGD treatment. Western blot analysis illustrated that miR-let-7i up-regulation increased the expression of Bcl-2, while miR-let-7i down-regulation suppressed its expression (Figure 4C). However, qRT-PCR analysis showed that the alterations in the expression level of miR-let-7i had no effect on the expression of Bcl-2 (Figure 4D). Overall, these results indicated that miR-let-7i positively regulated Bcl-2 at the post-transcriptional level.

## Discussion

Post-stroke cognitive impairment is an irreversible process that can directly lead to neuronal synaptic damage and dysfunctions of sensory, motor, and autonomic nerves (Jacquin et al., 2014; Trzepacz et al., 2015). Recently, it has been reported that the miRNAs are linked with synaptic plasticity and cognitive impairment (Saab and Mansuy, 2014). However, the potential mechanisms that these miRNAs may contribute to memory disorders have not been well declared. Meanwhile, given the limited diagnostic accuracy of existing biomarkers for PSCI, it is urgently needed to explore a novel diagnostic biomarker. Therefore, we enrolled a total of 74 patients with PSCI or non-PSCI to investigate the potential biomarker for PSCI and then investigate the mechanism of miR-let-7i in PSCI *in vitro*.

To our knowledge, this is the first report focused on the miR-let-7i in PSCI. In the present study, we firstly demonstrated that miR-let-7i was overexpressed in PSCI patients and it could be used as diagnostic biomarkers for PSCI with favorable diagnostic performance. Secondly, we illuminated the potential mechanism that miR-let-7i alleviated OGD-induced cell damage by positively regulating Bcl-2 at the post-transcriptional level.

It has been reported that the miRNA expression levels are consistent across individuals of the same species (Reid et al., 2011). Moreover, they present in human plasma in a remarkably stable form that protected them from endogenous RNase activity (Mitchell et al., 2008). Thus, miRNAs have the potential to be a novel diagnostic biomarker for disease diagnosis and developing new therapeutic interventions. In the present study, we confirmed the favorable diagnostic performance of miR-let-7i in PSCI, with an AUC of 0.859. Previously, Huang et al. (2016) have reported the excellent diagnostic performance of miR-132 in PSC with an AUC of 0.961. Additionally, miR-223 was also reported to be used as a diagnostic biomarker for acute ischemic stroke with an AUC of 0.859 (Chen et al., 2017). In summary, we believe that miRNAs are a potential diagnostic marker of PSCI. Future studies can be conducted to investigate the optimal diagnostic biomarkers for PSCI diagnosis.

The miR-let-7i is a member of the miRNA let-7 family, which has been widely researched in various tumors (Yang et al., 2008; Wu et al., 2015). In the present study, we investigate the mechanism of miR-let-7i in PSCI for the first time. Consistent with previous reports, we identified the overexpression of miR-let-7i in PCSI (Balakathiresan et al., 2012; Bhomia et al., 2016). Importantly, we demonstrated that miR-let-7i could protect cells from OGD-induced cell damage *in vitro* by targeting Bcl-2. Similarly, it was also reported to attenuate endothelial cell damage in the OGD model, which further supported our results (Xiang et al., 2017). Besides, multiple evidences have proved that miRNAs, such as miR-203, let-7, miR-195, and miR-122, all exert the anti-apoptotic effect to protect against cell injury (Yang et al., 2015; Guo et al., 2018; Han et al., 2018; Cheng et al., 2019). Thus, miRNAs have the potential to become the novel therapeutic target for nervous system disease.

Bcl-2 as an anti-apoptotic protein plays an important role in apoptosis regulatory (Vogler et al., 2017). In the present study, we observed the increase of Bcl-2 by the up-regulation of miR-let-7i. Previous studies have shown that Bcl-2 overexpression could protect against apoptotic cell death by inhibiting the activation of apoptosis-related proteins (Rohn et al., 2008). Thus, we assumed that miR-let-7i inhibited the activation of apoptotic pathways by targeting Bcl-2 expression. Besides Bcl-2, toll-like receptor 4 (TLR4) was identified as a target of miR-let-7i (Chen et al., 2007). It is widely demonstrated that TLR4 is involved in brain damage (Caso et al., 2007) and the knock-out of TLR4 has neuroprotective effects against cerebral ischemia–reperfusion injury (Pradillo et al., 2009; Hyakkoku et al., 2010). In experimental stroke, TLR4 is up-regulated in neurons as early as 1 h after stroke onset (Nagyőszi et al., 2010); meanwhile, in a clinical study, up-regulation of TLR4 has been found to be associated with poor stroke outcome (Zhang et al., 2012). Furthermore, Xiang et al. (2017) have reported that miR-let-7i could attenuate brain microvascular endothelial cell damage by decreasing TLR4 expression in the OGD model. The above evidences suggested that the up-regulation of miR-let-7i could alleviate the neuronal damage by targeting various regulatory factors, and it has the potential as a therapeutic target for PSCI. However, the molecular mechanisms have not yet been fully elucidated in the PSCI. Our study is based on *in vitro* studies, so there are still a few limitations and further study is required to test the hypothesis.

In summary, the miR-let-7i was verified to be overexpressed in PSCI patients and it could be used as diagnostic biomarkers for PSCI with an AUC of 0.859. Importantly, we illuminated the potential mechanism that miR-let-7i alleviated OGD-induced cell damage by positively regulating Bcl-2 at the post-transcriptional level. We believe that miR-let-7i has the potential as a biomarker and a therapeutic target for PSCI.

## Data Availability Statement

All datasets generated for this study are included in the article/supplementary material.

## Ethics Statement

The studies involving human participants were reviewed and approved by the Ethics Committee of the Cangzhou Center Hospital and Cangzhou People’s Hospital. The patients/participants provided their written informed consent to participate in this study.

## Author Contributions

P-YL and Z-QW: conceptualization. Z-QW and KL: methodology and writing – original draft preparation. JH: formal analysis. Z-QW and JH: resources. T-TH: data curation. JH and T-TH: writing – review and editing. P-YL: visualization, supervision, and project administration.

## Conflict of Interest

The authors declare that the research was conducted in the absence of any commercial or financial relationships that could be construed as a potential conflict of interest.

## Footnotes

1 http://www.targetscan.org
