# Corrigendum: MicroRNA Let-7i Is a Promising Serum Biomarker for Post-stroke Cognitive Impairment and Alleviated OGD-Induced Cell Damage *in vitro* by Regulating Bcl-2

**DOI:** 10.3389/fnins.2021.648121

**Published:** 2021-01-29

**Authors:** Zhan-Qiang Wang, Kuo Li, Jie Huang, Tian-Tian Huo, Pei-Yuan Lv

**Affiliations:** ^1^Department of Neurology, Hebei Medical University, Shijiazhuang, China; ^2^Department of Neurology, Hebei General Hospital, Shijiazhuang, China; ^3^Department of Neurology, Cangzhou People's Hospital, Cangzhou, China; ^4^No. 2 Department of Neurology, Cangzhou Central Hospital, Cangzhou, China

**Keywords:** miR-let-7i, microRNA, post-stroke cognitive impairment, Bcl-2, cell damage, oxygen–glucose deprivation

In the published article, there was an error regarding the affiliations for Zhan-Qiang Wang, Kuo Li, Tian-Tian Huo, and Pei-Yuan Lv. All of these authors are also affiliated to the Department of Neurology, Hebei Medical University, Shijiazhuang, China.

Additionally, there was an error in the original affiliation 3, now amended to be affiliation 4. Instead of “Department of Neurology, Cangzhou Central Hospital, Cangzhou, China” it should be “No. 2 Department of Neurology, Cangzhou Central Hospital, Cangzhou, China.”

The authors apologize for these errors and state that they do not change the scientific conclusions of the article in any way. The original article has been updated.

